# Cystic Adenoid Carcinoma: A Rare Bronchial Tumor

**DOI:** 10.7759/cureus.42476

**Published:** 2023-07-26

**Authors:** Afaf Thouil, Meriem Rhazari, Fatima Zahra Baddi, Hatim Kouismi

**Affiliations:** 1 Department of Respiratory Diseases, Research and Medical Sciences Laboratory, Faculty of Medicine and Pharmacy of Oujda, Mohammed VI University Hospital, Mohammed First University, Oujda, MAR; 2 Department of Pulmonology, Mohammed VI University Hospital, Oujda, MAR; 3 Department of Pulmonary, Centre Hospitalier Universitaire (CHU) Mohammed VI, Oujda, MAR

**Keywords:** bronchial mucosa, lung cancer, carcinoma, adenoid, cystic

## Abstract

Although very rare, cystic adenoid carcinoma (CAC) should be considered as a differential diagnosis for any lung tumor arising from the bronchial glands. The diagnosis is typically confirmed through histological examination, and treatment is primarily based on surgical intervention. In this report, we present the case of an 82-year-old male with primary CAC of the lung.

## Introduction

Primary cystic adenoid carcinoma (CAC) of the lung is a rare type of malignant epithelial tumor. It was previously referred to as cylindroma and interestingly, it does not show a preference for either gender. One of the key characteristics of CAC is its high resistance to radiation therapy, which makes surgical intervention the primary treatment approach. However, it's important to note that recurrence rates are quite high. This is largely due to early perineural invasion, which significantly contributes to these recurrences [1].

## Case presentation

**Figure 1:**
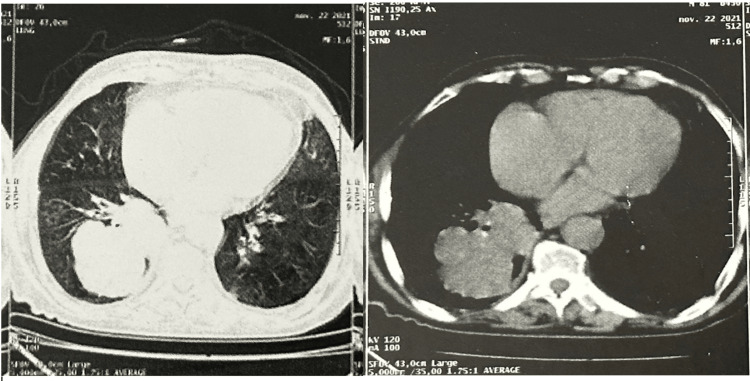
Chest CT scan showing a poorly delimited right post-basal segmental pulmonary lesion, suggestive of a possible malignancy. CT: computed tomography

We present the case of an 82-year-old patient with a previous history of chronic smoking, which ceased 40 years ago, and a cardiac condition that lacks documentation. The patient's chief complaints included chronic dyspnea, a dry cough, anorexia, and weight loss, without the presence of other respiratory or non-respiratory symptoms. Upon physical examination, no significant abnormalities were observed. A computed tomography (CT) scan revealed a confined lesion measuring 82 mm in the right posterior basal segment, with no evidence of secondary localization (Figure 1).

**Figure 2:**
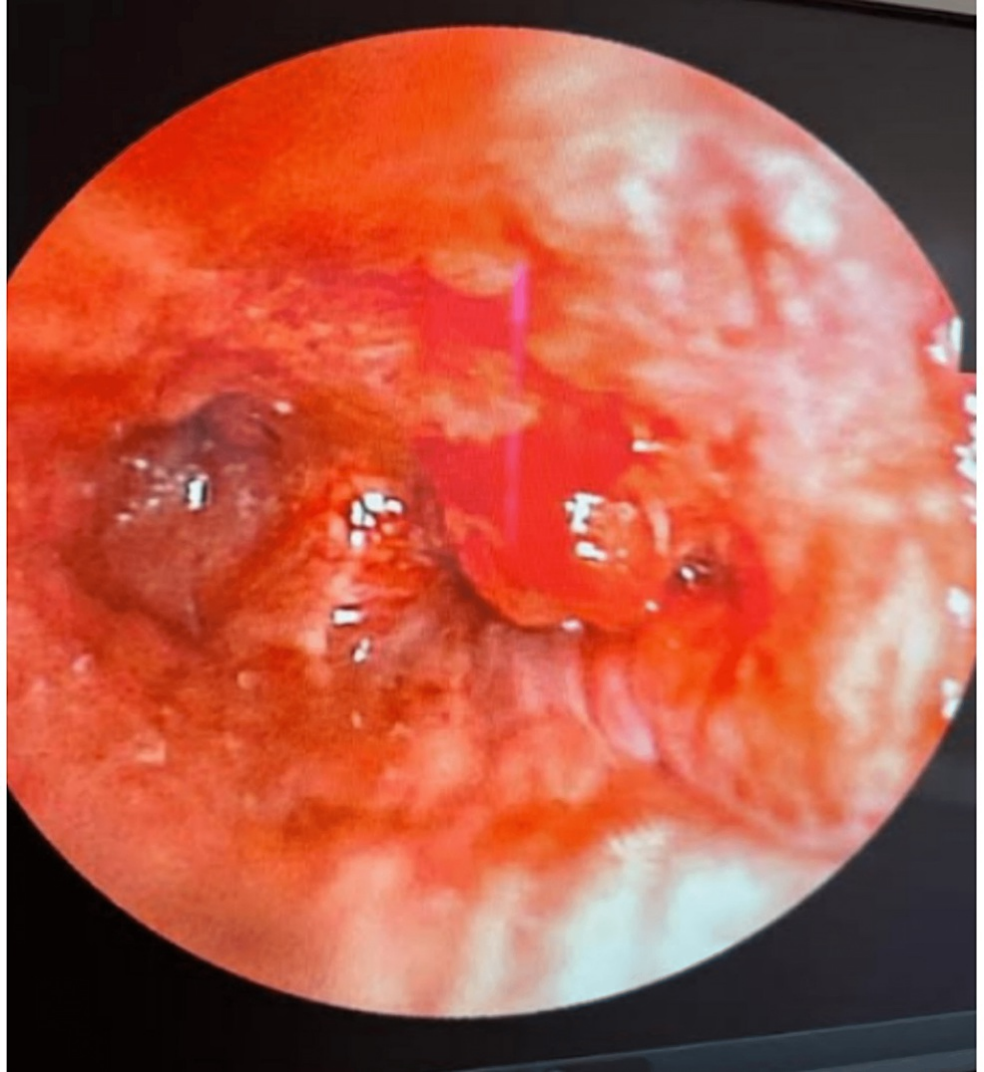
Bronchial fibroscopy showing infiltration of the bronchial mucosa at the basal pyramid with a complete obstruction at the entrance of the latter.

**Figure 3:**
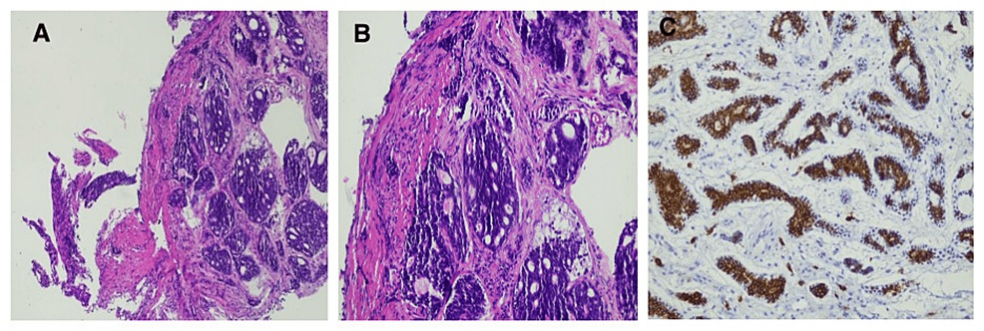
Histopathological and immunohistochemical features of bronchial carcinoma. (a) Microscopic image (HE x10) showing infiltration of bronchial mucosa by a carcinomatous tumor with cribriform growth pattern and fibrous stroma. (b) High magnification view (HE x20) showing eosinophilic material in the cribriform beds. (c) Immunohistochemistry image demonstrating expression of CD117 by tumor cells.

During bronchoscopy (Figure 2), a complete obstruction was identified at the entrance of the right basal pyramid. Subsequently, a bronchial biopsy was performed, and histological examination revealed a significant infiltration of the mucous membrane by a carcinomatous tumor proliferation. The tumor predominantly consisted of cribriform beds and glands, located within a fibrous and slightly inflammatory stroma (Figure 3). Notably, the cribriform beds contained eosinophilic material. The tumor cells exhibited large size, moderate anisonucleosis, and the presence of mitoses, with no observed perineural tumor involvement. Immunohistochemical analysis confirmed the diagnosis of primary CAC of the lung.

Examination of the ENT region, including the salivary glands, did not reveal any abnormalities. In our case, immediate surgical intervention was advised; however, regrettably, the patient declined the recommended treatment.

## Discussion

Primary lung tumors of the salivary gland type are rare but not exceptional and are predominantly represented by malignant entities such as CAC [1]. Pulmonary CAC constitutes less than 1% of primary lung tumors and typically develops intraluminally in the trachea or large bronchi, with potential extension into the pulmonary parenchyma or mediastinal fat [1,2]. Typically, it presents as an ill-defined tumor with small, scattered strands infiltrating the lung. The average age of patients is around 50 years, with no gender predominance or association with smoking [1-8]. In our case, the patient was an 82-year-old heavy male smoker. Clinical manifestations are predominantly characterized by signs of airway obstruction, including dyspnea, cough, chest pain, and hemoptysis [9,10]. Most commonly, this tumor arises in the trachea and stem or lobar bronchi [1,2,10]. It infiltrates the cartilage and adjacent peri-bronchial tissue [9,10]. Bronchoscopy can determine the location and extent of the tumor, but assessing the peri-bronchial tumor spread is challenging [2,10]. Chest CT scans are useful in identifying intraluminal, peritracheal, or peri-bronchial extension, as well as possible metastases [10]. Definitive diagnosis requires histological examination. CAC comprises epithelial and myoepithelial cells and exhibits three architectural variants: the cribriform form, characterized by cylindromatous masses and micro cysts filled with mucoid and basophilic hyaline material, which is the most common variant; the tubular form, consisting of tubes and ducts lined by double-layered internal epithelial and external myoepithelial cells; and the solid form composed of uniform basal cells. Tumors may be composite or have a predominant variant. Cytonuclear atypia and mitosis are infrequent. Staining with Alcian blue and periodic acid-Schiff (PAS) does not reveal mucosecretion. The tumor stroma can exhibit myxoid or fibrous and inflammatory features in some areas [1,3,7]. A classification system is applied to primary pulmonary CAC and salivary CAC, with tubular variants considered less aggressive. The prognosis remains guarded due to the malignant nature of the tumor and its potential for slow recurrence, typically occurring after seven to 15 years. Recurrences often result from inadequate tumor containment, as surgical margins may sometimes involve unhealthy areas. Therefore, performing intraoperative frozen section analysis to verify resection margins is recommended [1]. In cases of CAC, it is crucial to first rule out the possibility of metastatic salivary CAC and then differentially diagnose other primary lung tumors (adenocarcinoma, carcinoid tumor) or salivary epithelial carcinoma, including myoepithelial carcinoma. Based on the clinical and histological presentation of our patient, a primary lung tumor was suspected, and an ENT examination was conducted to exclude a primary tumor at that site. The mainstay of CAC treatment remains surgical intervention; however, complete resection is often challenging due to the infiltrative nature of the tumor along the airways [8,9,10]. A thorough examination of surgical margins is therefore essential [10]. In our case, following confirmation of malignancy, surgery was the recommended treatment option, although the patient declined.

## Conclusions

CAC of the lung is known for its slow growth yet high aggressiveness, often characterized by neural invasion. While it is most commonly found in the major and minor salivary glands, CAC can also occur in other areas, including the breast and lungs.
